# Ferroelectric nanodot reservoir for neuromorphic computing

**DOI:** 10.3762/bjnano.17.24

**Published:** 2026-02-20

**Authors:** Anna Razumnaya, Yuri Tikhonov, Dmitrii Naidenko, Léo Boron, Valerii Vinokur, Igor Lukyanchuk

**Affiliations:** 1 Condensed Matter Physics Department, Jožef Stefan Institute, Ljubljana, Slovenia; 2 Laboratoire de Physique de la Matière Condensée, Université de Picardie Jules Verne, Amiens, France; 3 International Research Institute for Intelligent Materials, Southern Federal University, Rostov-on-Don, Russia; 4 Terra Quantum AG, St. Gallen, Switzerland; 5 L. D. Landau Institute for Theoretical Physics, Moscow, Russia

**Keywords:** ferroelectric nanodots, multilevel logic, neuromorphic circuits, polarization, topological states

## Abstract

We present a ferroelectric-based data reservoir designed for neuromorphic computing applications. The reservoir consists of an ensemble of nonlinear ferroelectric nanodots capable of storing information through their stable polarization states. These nanodots are confined between electrodes and form a network of parallel connected capacitive elements, which can either be addressed individually or integrated between common conducting plates. By leveraging the intrinsic nonlinearity and memory of ferroelectric polarization switching, the device maps incoming signals into a high-dimensional space of polarization configurations. This physical transformation enables efficient temporal information encoding and provides a rich dynamic representation for subsequent processing in a readout layer of a neuromorphic circuit.

## Introduction

Neuromorphic computing aims to emulate the architecture and processing mechanisms of the human brain to achieve energy-efficient and adaptive information processing [1–6]. Among various neuromorphic approaches, reservoir computing has emerged as a particularly promising paradigm, owing to its conceptual simplicity and hardware amenability [7–11]. In reservoir computing, a high-dimensional, nonlinear dynamical system, called the reservoir, transforms input signals into a rich temporal multiplexed feature space, from which a linear readout extracts the target output for further neuromorphic processing. In recent years, significant efforts have been devoted to realizing physical implementations of reservoirs using diverse material platforms, including photonics [12], spintronics [13], and different types of memristors [14–15].

Ferroelectric materials offer a unique combination of properties (i.e., nonlinearity, nonvolatility, analog state tunability, and compatibility with CMOS platforms) that make them particularly attractive for energy-efficient neuromorphic circuits [16–21]. They inherently possess a multitude of metastable polarization orientations, corresponding to different local minima of the free energy [22–24]. This intrinsic multistability facilitates analog and digital information encoding, retention, and logic operations within a single physical unit, and is one of the key reasons for their growing relevance in neuromorphic computing and multivalued logic architectures [17,25]. Ferroelectrics also host a wide variety of nonuniform topological soliton-like polarization states, especially at the nanoscale [26–33]. The presence of such topological states provides not only robust multistability but also new functional degrees of freedom, including topologically protected switching, enhanced memory capacity, and localized polarization textures that can act as multilevel logic or signal-processing elements [34].

Ferroelectric field-effect transistors (FeFETs), ferroelectric tunnel junctions (FTJs), and memcapacitive synapses revealing ferroelectric properties, have been proposed and demonstrated as viable candidates for synaptic and reservoir elements [25,35]. In FeFET-based reservoirs, for example, the polarization-dependent hysteresis provides both the nonlinearity and memory required for temporal signal encoding [36]. Moreover, recent advances in material engineering have enabled reliable multilevel operation and subnanosecond switching speeds, which are essential for real-time neuromorphic processing [37]. A topologically configurable multilevel logic unit based on interacting ferroelectric domains proposed in [38–39] showed how polarization states can be harnessed for logic operations within a compact physical structure.

While various approaches have demonstrated the use of ferroelectrics in neuromorphic computing at the level of individual elements, most existing implementations treat each ferroelectric component as an isolated device or a deterministic node within a predefined circuit. However, such approaches often neglect the rich collective dynamics emerging from ensembles of interacting nonlinear elements, an aspect especially compelling when analyzed through the lens of statistical physics models such as the Ising spin system with disorder. Harnessing the emergent behavior of ferroelectric interconnected nanostructures as physical reservoirs thus represents a largely underexplored yet potentially transformative direction for neuromorphic computing.

In this work, we present a ferroelectric-based data reservoir concept grounded in the collective nonlinear response of an ensemble of ferroelectric nanodots. The system consists of a network of nanodots, each carrying the bistable polarization state, arranged between electrodes in configurations that allow for parallel electrical addressing. By receiving temporal input signals, the ensemble evolves into distinct polarization states that encode input history through a combination of nonlinear switching and spatial interactions. This physical reservoir naturally maps the input signal into a high-dimensional space, making it suitable for neuromorphic inference tasks.

The objective of this work is to establish the physical principles and theoretical framework of a charge-driven ferroelectric reservoir based on nanodot ensembles. We delineate the mechanisms that endow the system with nonlinearity, and high dimensionality, linking electrostatic coupling, hysteretic switching, and the resulting frustrated-Ising energy landscape, and outline practical interface schemes for signal injection and readout. Altogether, the presented framework connects the collective polarization dynamics of ferroelectric nanodots with emergent nonlinear and memory effects, providing a solid foundation for developing and engineering compact, energy-efficient, and scalable ferroelectric platforms for neuromorphic information processing.

The objectives of this work are to introduce a physically realizable reservoir architecture based on ferroelectric nanodots, to highlight the mechanisms enabling nonlinearity, fading memory, and high dimensionality in such a system, and to position this architecture relative to existing approaches in neuromorphic hardware. Our findings suggest that ensembles of ferroelectric nanodots offer a compact, low-power, and scalable solution for implementing physical reservoirs with emergent computational capabilities.

## Results and Discussion

### General concept

**Figure 1:**
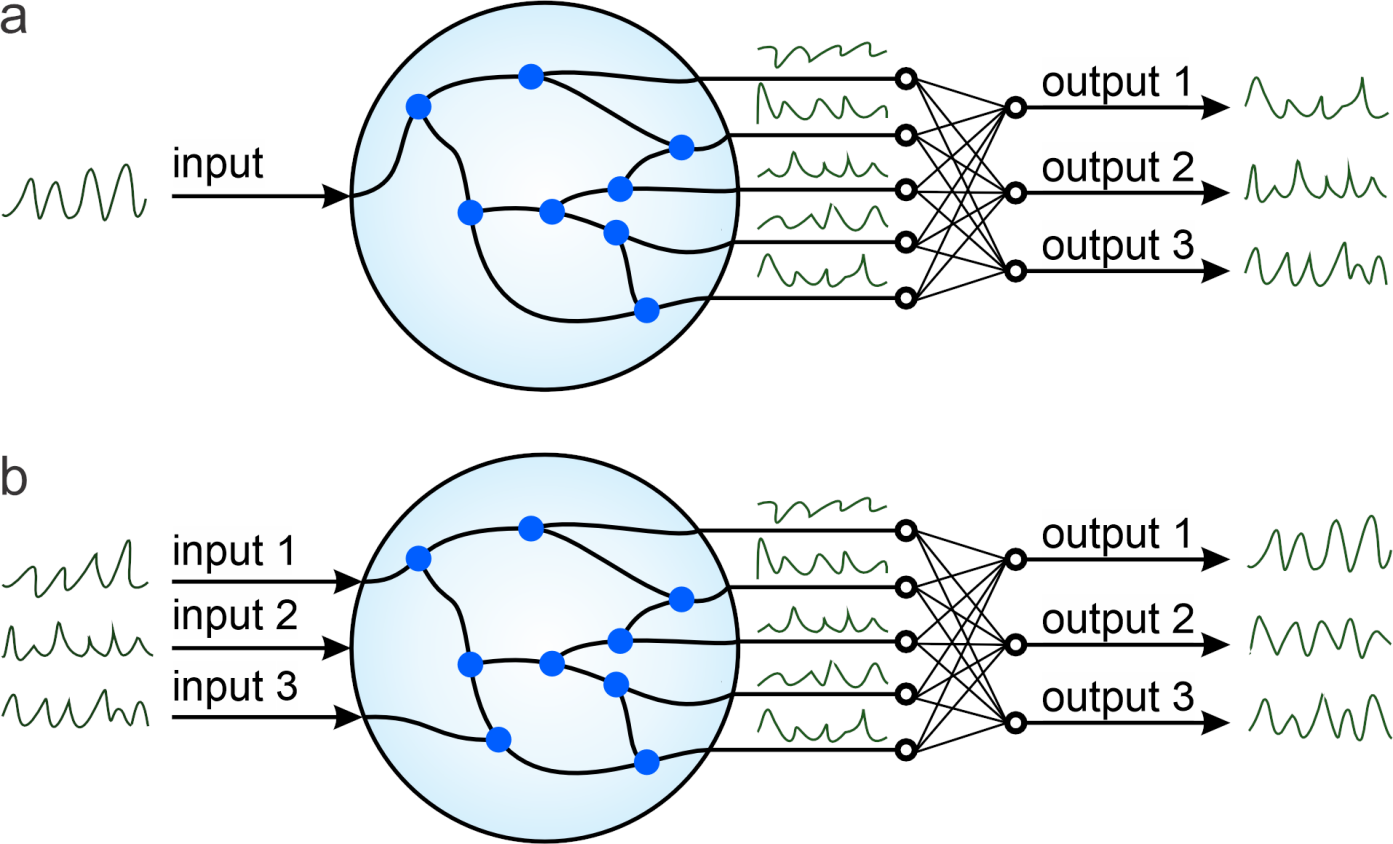
The reservoir computing system consists of an array of ferroelectric nanodots (blue points) forming an ensemble of capacitors that are electrically connected either through a network of wires or via common electrodes. (a) Configuration with a single input channel integrated into the neuromorphic circuit. (b) Configuration with multiple input channels integrated into the neuromorphic circuit.

The proposed reservoir architecture is designed to physically implement the principles of reservoir computing using the interconnected ferroelectric nanodots as nonlinear memory-storage nodes. The system enables efficient temporal information processing by exploiting polarization dynamics under external input signals. Figure 1 illustrates two conceptual configurations of the ferroelectric-based reservoir and its integration into a neuromorphic computing framework.

In Figure 1a, a single input channel delivers time-dependent electrical or optical signals into the ferroelectric reservoir. The reservoir consists of an ensemble of nanodots, each exhibiting a nonlinear bistable polarization response ±*P*_s_(*i*) (where *i* is the nanodot index and *P*_s_ is the magnitude of the spontaneous polarization) to external excitation. The ensemble of these nanodots forms a network of parallel connected capacitors, either through a wired interconnection or via a shared common electrode. Upon receiving the common input signal *x*(*t*), the polarization states of the nanodots evolve collectively, producing a high-dimensional internal state. This internal state is projected onto the readout layer, which samples the states of a selected subset of nanodots *j* and performs linear operations (e.g., weighted summation) to generate the multidimensional system output *y_j_*(*t*).

In Figure 1b, a more general case is presented, where multiple input electrical channels *x_j_*(*t*) feed the reservoir simultaneously. These can represent different signal modalities, spatially distributed sensors, or temporally multiplexed spike trains. The multichannel configuration allows the reservoir to integrate and correlate complex spatiotemporal patterns, offering an increase in expressive power and applicability for multidimensional signal processing tasks.

Conceptually, such architectures align with theoretical foundations established in traditional reservoir computing frameworks. However, unlike systems relying on sequential switching or delay-based dynamics, the proposed design enables true parallel evolution of polarization states with low energy consumption and fast switching speeds, leveraging the intrinsic properties of ferroelectrics.

Furthermore, while previous considerations of neuromorphic ferroelectric nanodot-based setups [38–39] demonstrated static logic functions in topologically structured ferroelectric domain networks, the present system shifts toward dynamic, time-dependent processing, essential for tasks such as time-series prediction, temporal pattern recognition, and classification. The reservoir’s ability to encode temporal correlations through physical polarization evolution distinguishes it from traditional logic-oriented implementations and brings it closer to biologically inspired neural architectures.

The distinction between the single- and multiinput configurations shown in Figure 1 reflects the system’s scalability and adaptability. In real-world neuromorphic systems, multiple input streams are often required to represent multimodal sensory information or parallel processing pipelines. The ability of ferroelectric reservoirs to naturally support such integration without increasing circuit complexity presents a significant advantage over transistor-based logic systems.

### Core nanodot unit

**Figure 2:**
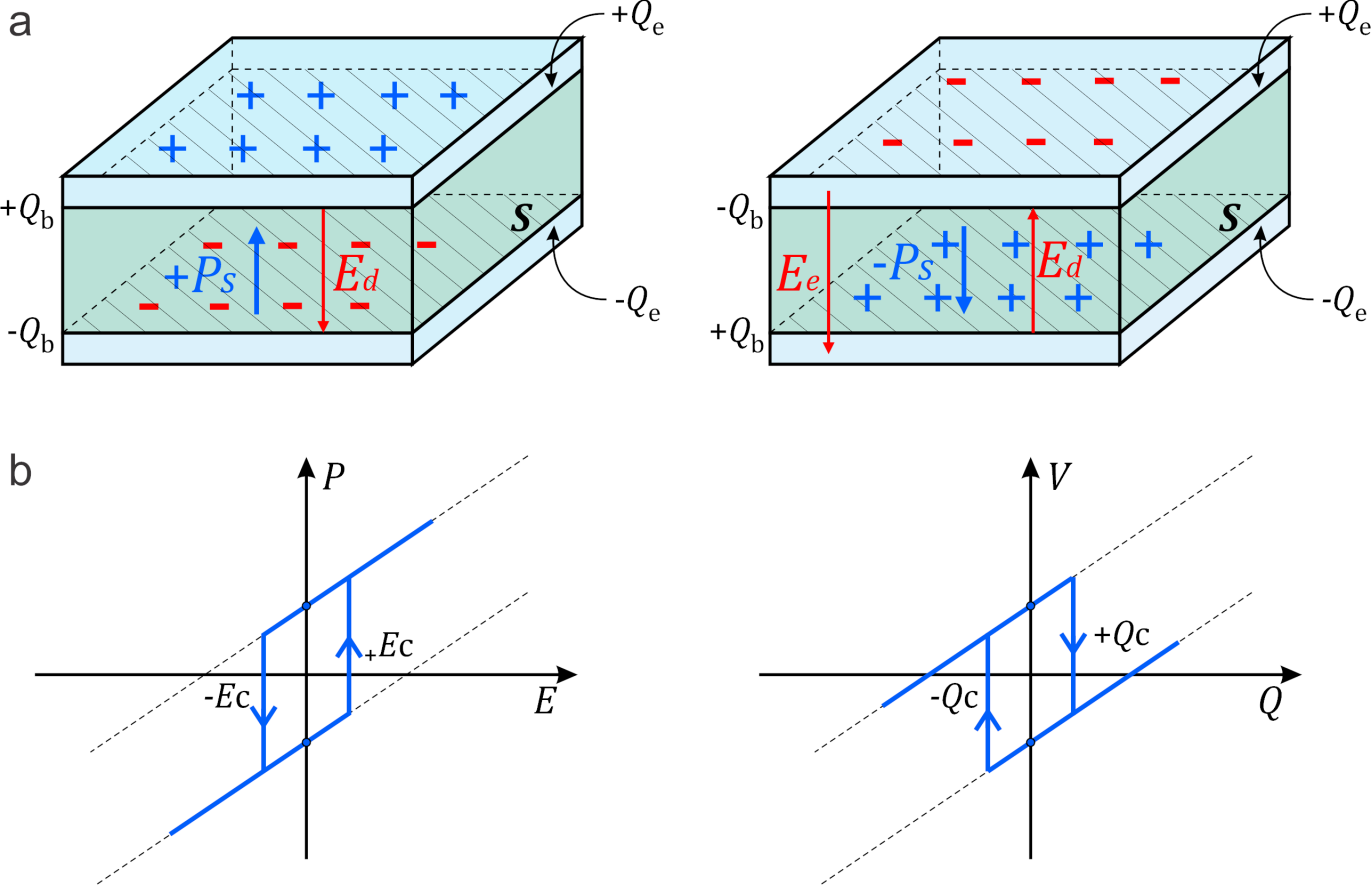
The basic operations of a single ferroelectric nanodot. (a) Schematic of a ferroelectric nanodot placed between two conducting plates, representing the constitutive unit of the ferroelectric reservoir. (b) Polarization–electric field *P*(*E*) and voltage–charge *V*(*Q*) hysteresis loops characteristic of ferroelectric switching.

To describe the basic operational mechanism of the ferroelectric reservoir introduced in Figure 1, we now examine its elementary building block, the ferroelectric nanodot. Figure 2 illustrates the structure and the nonlinear switching behavior of a single nanodot under external electrical excitation.

As shown in Figure 2a, the nanodot is confined between two metallic electrodes. It exhibits a spontaneous polarization *P*_s_ with at least two stable orientations: “up” and “down”. These polarization states are denoted as *mP*_s_ where *m* = +1 corresponds to the “up” state and *m* = −1 to the “down” state. Termination of polarization at the top and bottom surfaces results in bound charges of magnitude *mQ*_b_ = *mSP*_s_, where *S* is the cross-sectional area of the nanodot. These surface-bound charges produce an internal depolarization field, *E*_d_ = −*mP*_s_/ε_0_ε_f_, where ε_0_ is the vacuum dielectric permittivity and ε_f_ is the dielectric constant of the ferroelectric material. An external charge ±*Q*_e_ applied to the electrodes generates an additional electric field inside the nanodot, *E*_e_ = −*Q*_e_/ε_0_ε_f_*S*. Therefore, the total electric field inside the nanodot is the sum of the depolarization field, *E*_d_, and the electric field produced by the conducting plates, *E*_e_, and can be written as *E* = −(*mP*_s_*S* + *Q*_e_/*S*)/ε_0_ε_f_.

The corresponding electrostatic energy stored in the nanodot of thickness *d* is:


[1] $$U=\frac{1}{2}\varepsilon_{0}\varepsilon_{\text{f}}E^{2}Sd=\frac{{\left(mP_{s}+Q_{e}/S\right)}^{2}}{2\varepsilon_{0}\varepsilon_{\text{f}}}\cdot Sd.$$

This energy has two local minima corresponding to the two polarization orientations, and the system tends to settle in the one with lower energy under the given external charge. That is, for *Q*_e_ *<* 0, the “up” state (*m* = +1) is energetically favorable, for *Q*_e_ *>* 0, the “down” state (*m* = −1) is preferred. Notably, the switching between these states occurs via a nonlinear, hysteretic transition. This transition is governed by the coercive electric field *E*_c_. The coercive charge required for switching is: *Q*_c_ = ε_0_ε_f_*SE*_c_, where the positive value corresponds to switching from “up” to “down”, and the negative value corresponds to the reverse process. This behavior is illustrated in Figure 2b, showing typical hysteresis loops for both polarization vs electric field *P*(*E*) and voltage vs charge *V*(*Q*).

These hysteresis curves realize the bistability and nonlinearity that are central to the function of the reservoir. Similar behaviors have been experimentally verified in a variety of ferroelectric materials, such as HfO_2_-based FeFETs [36], and Pb(Zr,Ti)O_3_ or BiFeO_3_ nanostructures [16–17]. In addition to their bistable switching behavior, ferroelectric nanodots can exhibit negative capacitance under specific conditions, particularly during polarization reversal processes. This regime, where an increase in voltage leads to a decrease in charge, arises from the energy-lowering feedback between polarization and internal electric field. Negative capacitance not only enhances the effective electrostatic tunability of the system but also provides a potential route to voltage amplification and energy-efficient signal processing at the nanoscale [40–41]. Incorporating such effects into the reservoir architecture could further enrich its nonlinear dynamics and expand the range of neuromorphic functionalities.

### Networking of ferroelectric nanodots

The total reservoir is realized by assembling a network of such nanodots between shared or interconnected conducting plates, enabling electrical connectivity and collective dynamics among nodes. This configuration allows for the emergence of spatiotemporal polarization patterns, which can encode input histories and project them into high-dimensional internal states. This architecture goes beyond prior logic-function-based designs [38–39], enabling dynamic computation within a neuromorphic reservoir framework. Such physically grounded bistable elements correspond to the neurons in traditional recurrent neural networks or echo state networks [7,42], offering energy-efficient, scalable, and high-speed alternatives to digital implementations of reservoir computing.

**Figure 3:**
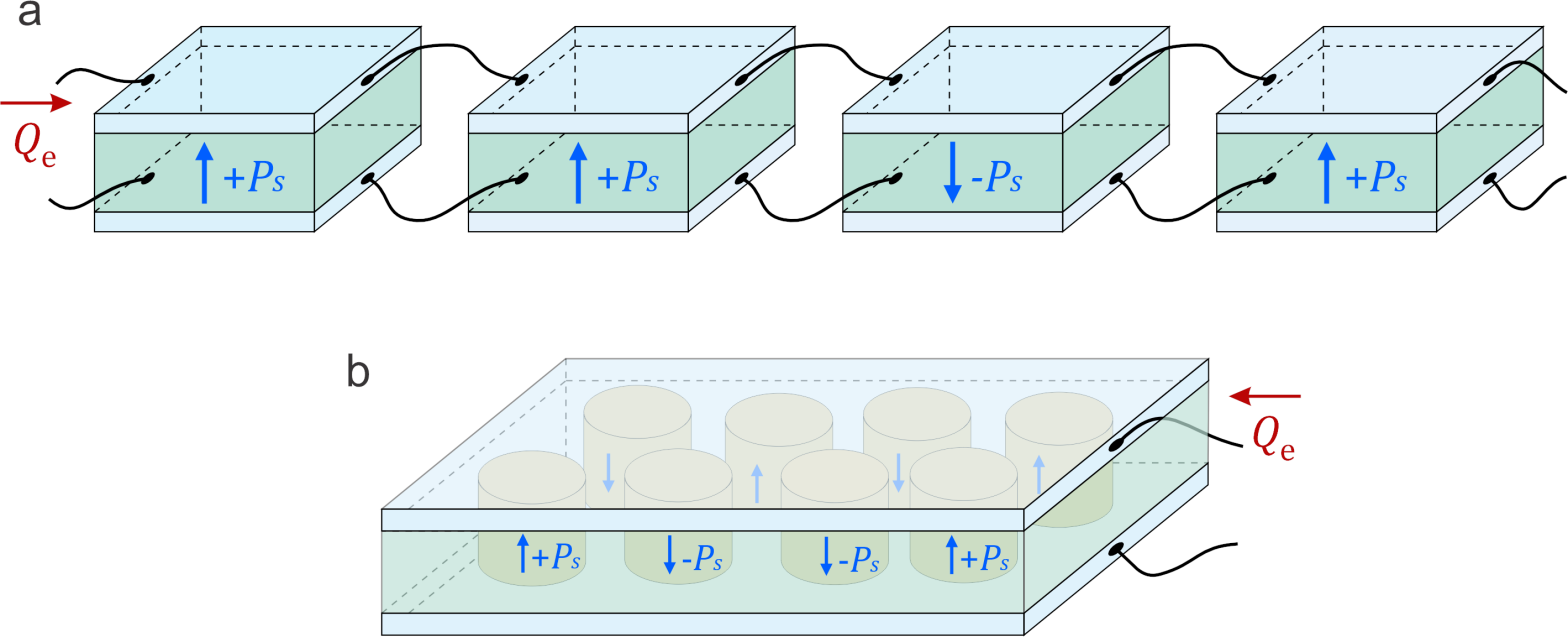
A schematic construction of a ferroelectric reservoir operated under charge-controlled conditions, with the injected charge *Q*_e_ represented by the arrow showing charge input at the electrode. (a) Ferroelectric reservoir realized as a network of wire-connected capacitors, each containing a single nanodot. (b) Ferroelectric reservoir in which all nanodots are confined between two common electrodes.

Figure 3 illustrates two exemplary charge-driven network architectures in which the total external charge *Q*_e_ applied to the electrodes governs the internal electric field and polarization state of the nanodots. In Figure 3a, each ferroelectric nanodot is embedded in an individual capacitor structure, and the capacitors are electrically connected in parallel through metallic interconnects. In Figure 3b, an alternative layout is shown in which all nanodots share the same pair of extended electrodes. In this case, the nanodots are arranged between two continuous conductive plates, with a dielectric filler possibly present between adjacent units.

Both configurations presented in Figure 1 can be described as an array of capacitors connected in parallel, where each nanodot represents an individual binary unit *i* with a specific polarization orientation, either “up” or “down”, denoted as *m_i_* = ±1. The corresponding bound charges on the surfaces of the nanodots are equal to *Q*_b_*_i_* = *m_i_P*_s_*S_i_*, where *S_i_* is the cross-sectional area of the *i*-th nanodot. The total free charge, *Q* = ±Σ*_i_P*_s_*S_i_m_i_* ± *Q*_e_, redistributes over the electrodes to maintain equipotentiality, ensuring that the internal electric field across each nanodot, *E* = −*Q*/ε_0_ε_f_*S*, is uniform. Here ±*Q*_e_ is the externally applied charge and *S* = Σ*_i_S_i_* is the total electrode area. The condition of the uniformly constant electric field throughout the array is a crucial feature for simultaneous control of polarization dynamics across nanodots.

The total electrostatic energy of the reservoir system becomes:


[2] $$U=\frac{1}{2}\varepsilon_{0}\varepsilon_{\text{f}}E^{2}Sd=\frac{1}{2}\sum_{ij}J_{ij}m_{i}m_{j}+\mu \sum_{i}h_{i}m_{i}+U_{0}.$$

Here, the system assumes the form of a frustrated Ising model with long-range antiferromagnetic interactions. The first term describes interactions between all pairs of sites via:


[3] $$J_{ij}=\frac{SdP_{\text{s}}^{2}}{\varepsilon_{0}\varepsilon_{\text{f}}}\cdot \frac{S_{i}S_{j}}{S^{2}}.$$

The second term captures the interaction of local polarization with an effective dipolar field, where:


[4] $$µ=P_{\text{s}}d,\;h_{i}=\frac{Q_{e}}{\varepsilon_{0}\varepsilon_{\text{f}}}\cdot \frac{S_{i}}{S}.$$

The final term *U*_0_ is independent of the configuration {*m_i_*}. Because all *J_ij_* are positive, the system is highly frustrated, meaning it has many nearly degenerate states near the energy minimum, a property that enables high information storage and computational richness. Such statistical properties are closely related to neuromorphic architectures. In analogy with the brain, each nanodot (site) functions similarly to a binary neuron, being in an “active” or “inactive” state, corresponding to *m_i_* = +1 and −1, respectively. The overall system thus mimics the dynamics of a large neural ensemble, offering capabilities for associative memory, pattern recognition, and temporal processing, consistent with the goals of physical reservoir computing.

A key feature of this reservoir is that the polarization switching at each site is hysteretic. The local coercive field at site *i* is expressed as:


[5] $$h_{\text{c}i}=\frac{Q_{\text{c}i}}{\varepsilon_{0}\varepsilon_{\text{f}}}\cdot \frac{S_{i}}{S}.$$

where *Q*_c_*_i_* is the site-specific coercive charge. This introduces an additional layer of dynamic complexity into the system.

To illustrate the reservoir behavior, consider a simplified case: All nanodots have equal area *S_i_* = *S*/*N*, but different coercive thresholds *h*_c_*_i_*. In this case, the interactions become homogeneous:


[6] $$J_{ij}=J=\frac{SdP_{\text{s}}^{2}}{N^{2}\varepsilon_{0}\varepsilon_{\text{f}}},\;h_{i}=h=\frac{Q_{\text{e}}}{N\varepsilon_{0}\varepsilon_{\text{f}}}.$$

The energy simplifies to:


[7] $$U=\frac{1}{2}J\sum_{ij}m_{i}m_{j}+\mu h\sum_{i}m_{i}+\text{const}.$$

Such a system has a high degree of degeneracy. At zero applied charge *Q*_e_ = 0, the minimum energy of the system corresponds to the state with zero total polarization, where half of the nanodots have up-directed polarization and the other half have down-directed polarization. For an even number *N* of nanodots, the degeneracy of this state is given by 
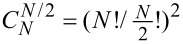
. If a positive charge increment *q* = *J*/*h* is applied, it flips the polarization of one nanodot from down to up, yielding a new configuration with *N*/2 − 1 nanodots oriented downward and *N*/2 + 1 upward. The degeneracy of this excited state is:


[8] $$C_{N}^{N/2-1}=\frac{N!}{\left(\frac{N}{2}-1\right)!\left(\frac{N}{2}+1\right)!}.$$

More generally, for *n* units of positive charge (with integer *n < N*), the resulting degeneracy becomes:


[9] $$C_{N}^{N/2-n}=\frac{N!}{\left(\frac{N}{2}-n\right)!\left(\frac{N}{2}+n\right)!}.$$

This exponential multiplicity of nearly degenerate states constitutes a powerful computational feature of the ferroelectric reservoir, enabling rich dynamic response to time-varying inputs, as required for real-time neuromorphic tasks. Notably, our approach leverages the electrostatic physics of nanoscale ferroelectrics to implement a scalable, low-power analog computing substrate. The described system relies solely on dielectric polarization degrees of freedom, making it especially attractive for dense and passive neuromorphic computing elements.

### Collective switching dynamics

**Figure 4:**
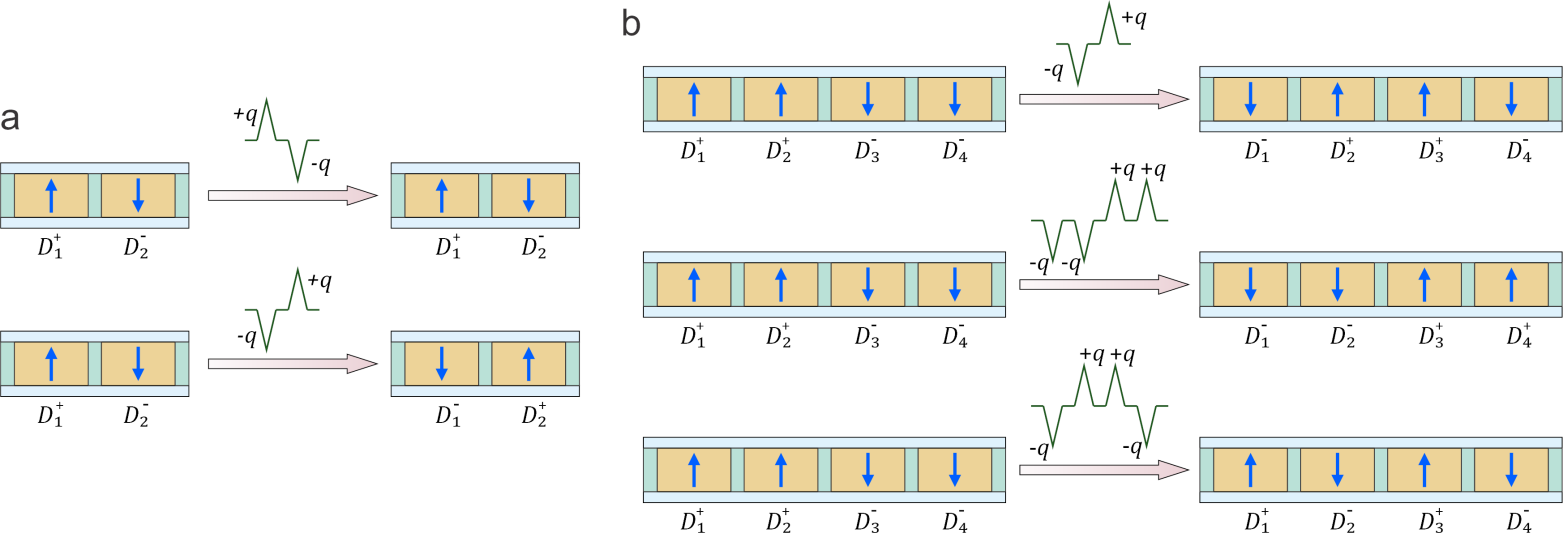
Switching behavior in ferroelectric reservoirs with a small number of nanodots driven by charge pulses. Evolution of the polarization states in (a) a two-nanodot reservoir under different charge pulse sequences and (b) a four-nanodot reservoir under different charge pulse sequences.

Figure 4 illustrates the possible variety of transitions within small-scale ferroelectric reservoirs comprising nanodots with different coercive fields, in response to various sequences of electrical charge pulses. These transitions exemplify the non-commutative and history-dependent behavior intrinsic to the system, stemming from the interplay of electrostatic interactions and local switching thresholds.

Figure 4a depicts a two-nanodot system with an initial configuration (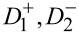), where the superscripts “+” and “−” denote up- and down-oriented polarization, respectively, and the subscripts label the associated coercive fields *h*_c1_ *< h*_c2_. Under the application of a positive charge unit +*q*, the nanodot 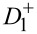, having the lowest coercive field, undergoes polarization reversal, yielding the intermediate state (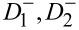). Upon applying a subsequent negative pulse *−q*, the same nanodot reverts to its original up-polarized state, restoring the initial configuration. Thus, the pulse sequence (+*q*,−*q*) leads to a reversible switching cycle:




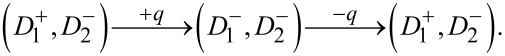




In contrast, reversing the order of pulses produces a distinct final state. Starting from (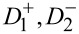), applying −*q* flips 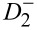, resulting in (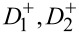). Then, +*q* switches 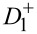 to (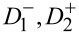). The final configuration thus depends on the order of the applied pulses:




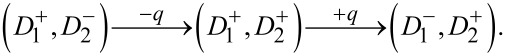




This demonstrates the non-commutative behavior of polarization dynamics in the reservoir, arising from the hierarchy of coercive fields. Figure 4b extends this analysis to a four-nanodot system with initial configuration (
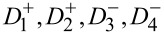
), and coercive fields ordered as *h*_c1_ *< h*_c2_ *< h*_c3_ *< h*_c4_. Here, multiple pulse sequences lead to distinct final polarization states depending on their magnitude and order.

The sequence (−*q*,+*q*) results in




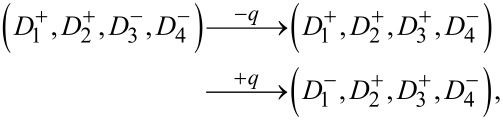




whereas the sequence (−2*q*,+2*q*) results in




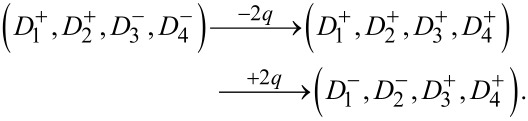




Finally, the sequence (−*q*,+2*q*,−*q*) results in




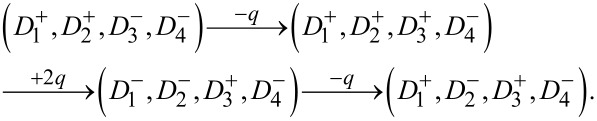




Here, each pulse of magnitude *q* denotes a unit of applied charge sufficient to flip the polarization of a nanodot with the lowest coercive field among those opposing the applied field. A double pulse of magnitude 2*q* corresponds to either a single pulse of double strength or two consecutive unit pulses, (2*q*) = (+*q*,+*q*). The results emphasize the resetting behavior of specific sequences. For instance, a sequence (+2*q*,−2*q*) can drive the reservoir first into a fully down-polarized state (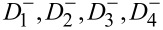), and then reinitialize it back to the original configuration (
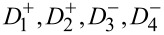
). Likewise, the inverse sequence (−2*q*,+2*q*) results in full up-polarization, followed by relaxation into a new asymmetric state (
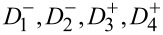
). These examples highlight the rich state transition dynamics available in ferroelectric reservoirs due to the combination of hysteresis, coercive field inhomogeneity, and global electrostatic interactions. Such dynamics provide the essential nonlinear temporal memory required for time-dependent information processing in neuromorphic systems.

### Signal processing

**Figure 5:**
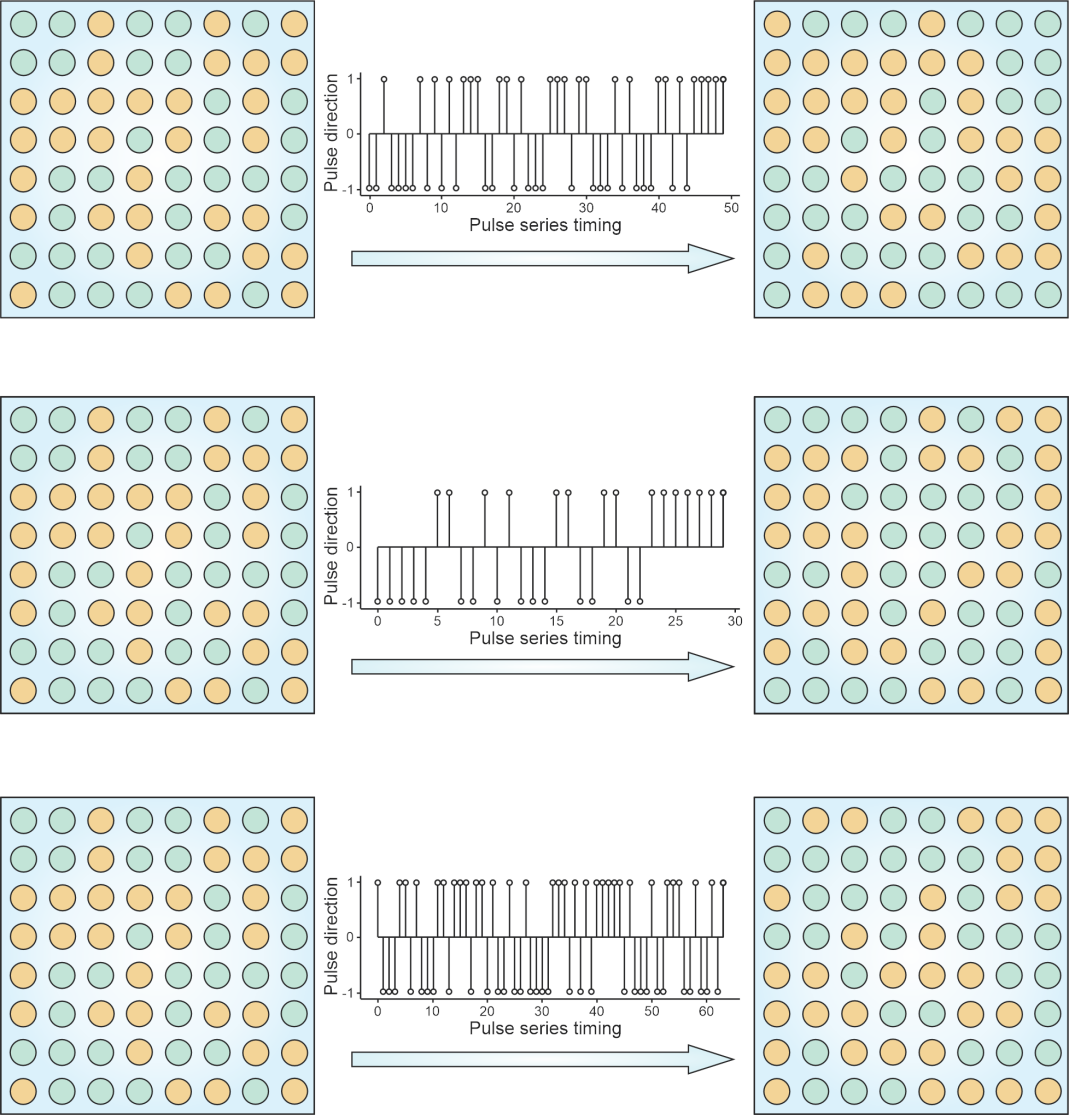
Dynamic evolution of polarization configurations in a square array of 256 ferroelectric nanodots with uniform cross-sectional area and randomly distributed coercive fields *h*_c_*_i_*, subjected to varying sequences of charge pulses. The reservoir is initialized by a reset pulse sequence (+*nq*,−*nq*), with *n* = 132, and subsequent signal pulses reconfigure the polarization pattern. The complexity and exponential state space of the system illustrate its suitability for high-dimensional temporal information processing in neuromorphic circuits.

We now turn to large systems where the collective behavior of many interacting elements enables rich spatiotemporal dynamics. We illustrate this behavior by simulating a square *N*^2^ = 16 × 16 ferroelectric nanodot array arranged in a square lattice, as shown in Figure 5. Each nanodot in the array shares the same geometry but differs in its coercive field *E*_c_*_,i_*, which is randomly assigned to reflect intrinsic material inhomogeneity. These coercive fields are taken from a static random distribution that remains fixed throughout the simulation, emulating quenched disorder and the inherent variability of switching thresholds in nanoscale ferroelectrics. The polarization state of each dot is binary, taking either +1 (“up”) or −1 (“down”). Switching occurs when the applied electric field *E* exceeds the local threshold *E*_c_*_,i_*.

After assigning the static coercive-field distribution, the simulation begins with a reset protocol: Alternating bipolar charge pulses (+*nq*, −*nq*) with *n* = [*N*^2^/2] = 132 are applied through common top and bottom electrodes, initializing the array into an initial reproducible state. Then, during signal processing, the field *E* is allowed to evolve in discrete steps, randomly increasing or decreasing by one unit, within a bounded range (−*n* to +*n*). At each step, the dot with the lowest switching threshold among the eligible ones undergoes a polarization reversal, thereby updating the system state.

This discrete event simulation captures the nonlinear hysteretic evolution of the entire reservoir. Importantly, because of the broad distribution of *E*_c_ and the collective nature of switching, the system explores a high-dimensional space of metastable configurations. The response depends not only on the current field but also on the sequence of past stimuli, a hallmark of temporal memory.

Each time the system returns to a balanced state with an equal number of +1 and −1 polarizations, the corresponding field trajectory is saved. These trajectories encode distinct input histories and illustrate how the reservoir transforms temporal sequences into spatial polarization patterns. Such behavior enables robust nonlinear signal processing and supports the implementation of neuromorphic tasks, including classification, prediction, and temporal pattern recognition.

This type of driven evolution has several profound consequences for neuromorphic reservoir operations: (i) Nonlinear input mapping. Due to the broad distribution of coercive thresholds, even small changes in the input sequence (field trajectory) can cause large and qualitatively different polarization responses. This sensitivity enables powerful separation of input classes in the reservoir state space. (ii) Short-term memory. The system retains a history of past field variations through its current polarization configuration. This memory is distributed and may decay over time due to charge leakage, mimicking the fading memory property of biological neural networks. (iii) High-dimensional projection. The number of accessible metastable states scales exponentially with the number of nanodots, ensuring that even simple temporal inputs are transformed into complex, spatially structured responses that can be linearly separated by a readout layer. (iv) History dependence and non-commutativity. Because switching paths depend on the temporal order of inputs, the same final field value may lead to different system states. This context-dependent behavior is vital for tasks such as temporal pattern recognition and sequence classification. In the present study, we focus on the intrinsic physical dynamics of the ferroelectric reservoir, which performs the major part of nonlinear preprocessing. Consequently, the associated readout layer can remain minimal, typically linear, while the principal feature transformation occurs within the reservoir itself.

### Interface architecture

The setup considered in the previous section corresponds to the case where all nanodots are confined between the same pair of conducting electrodes, creating a global capacitive structure. Also, similar input-output behavior and dynamic configurability can be realized in an alternative configuration, where each ferroelectric nanodot is embedded in an individual parallel-connected capacitor, as shown earlier, in Figure 3a. In both scenarios, the incoming data pulses are injected as electrical signals (e.g., current or voltage spikes) and dynamically reconfigure the reservoir state by switching local polarizations.

The readout layer, responsible for interpreting the reservoir’s final polarization state, may be connected to synaptic circuits that transduce polarization configurations into output signals for downstream neuromorphic processing. This integration strategy supports both dense analog information storage and real-time parallel processing. Although several previous works have modeled large-scale Ising-type networks for neuromorphic purposes using abstract or spin-based physical systems [43–44], the ferroelectric platform described here uniquely combines analog configurability, scalability, and low-energy operation due to the intrinsic dielectric polarization physics. In contrast to spintronic or CMOS-based implementations, the ferroelectric reservoir utilizes no active current-driving elements, allowing for passive, high-density neuromorphic integration.

**Figure 6:**
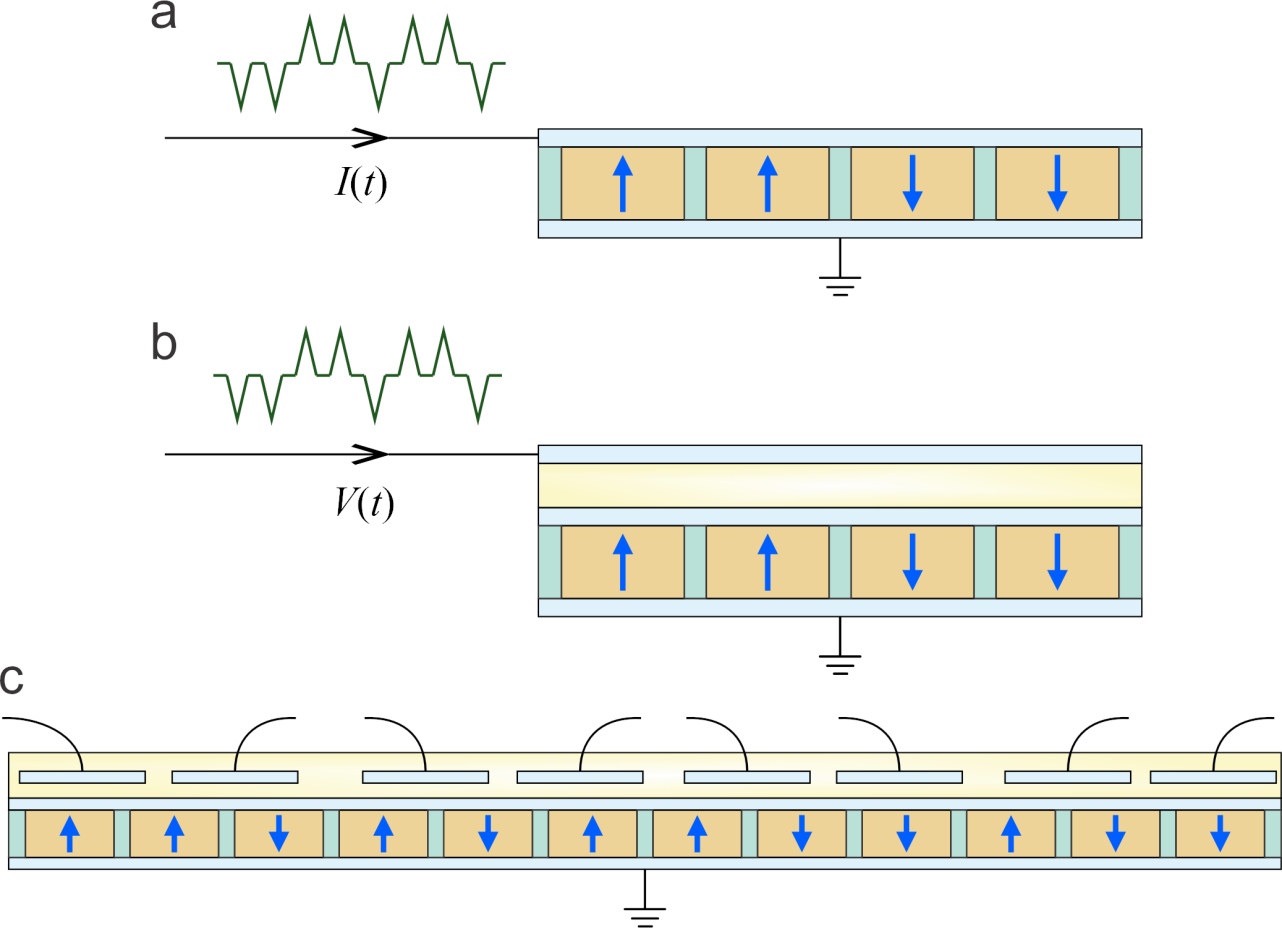
Signal injection and readout schemes for the ferroelectric reservoir. (a) Time-integrated current produces charge pulses for driving the reservoir. (b) Time-dependent voltage applied across a dielectric-reservoir capacitor pair enables voltage-controlled charge input and nonlinear readout. (c) Spatially multiplexed data is injected via spot electrodes and read out through similar localized outputs.

To interface the ferroelectric reservoir with external neuromorphic systems, specific schemes for signal injection and readout are required. Several architectures illustrated in Figure 6 demonstrate the versatility of input/output integration mechanisms compatible with ferroelectric reservoir operation.

In the scheme shown in Figure 6a, the input dataset is time-multiplexed into a series of current pulses or a continuous current signal *I*(*t*), which is applied to the upper electrode of the ferroelectric reservoir. The time-integrated current produces a sequence of charge pulses *Q*(*t*) = ∫*I*(*t*)d*t*, effectively driving the reservoir with a controlled temporal charge profile. The lower electrode is grounded. This setup ensures that the data is encoded as variations in total driving charge, enabling the dynamic evolution of polarization states across the nanodot network in response to temporal input patterns.

An alternative implementation, depicted in Figure 6b, implements a voltage-driven input by connecting the reservoir capacitor *C*_r_ in series with a smaller dielectric capacitor *C*_d_. A time-dependent voltage signal *V*(*t*), composed of pulses or a continuous waveform, is applied to the entire structure. If *C*_d_ ≪ *C*_r_, then most of the voltage drop occurs across the dielectric capacitor, and the charge delivered to the reservoir electrode is approximated by *Q*(*t*) = *C*_d_*V*(*t*). This configuration effectively converts the voltage signal into a corresponding charge signal, allowing for reservoir driving through voltage control. Moreover, the time-dependent voltage at the reservoir electrode, resulting from internal polarization dynamics, can be monitored as a readout signal. Since the voltage response reflects complex nonlinear processes such as switching and relaxation, it serves as a rich source of output features, analogous to synaptic signals in neuromorphic circuits.

A further signal integration scheme, illustrated in Figure 6c, uses spatial multiplexing, where individual data units are injected into specific locations of the reservoir via an array of small spot electrodes placed above the common charge-driving electrode. These local input electrodes modulate the nearby charge distribution, which in turn alters the polarization states of underlying ferroelectric nanodots. For readout, a similar array of spatially resolved electrodes senses the local electric fields that reflect the polarization configuration of the reservoir. These electrodes can be directly interfaced with synaptic elements in a downstream neural layer, enabling spatially structured output. In practical implementations, the readout vector is constructed from either local voltage responses of selected nanodots or from the global polarization configuration, which can be directly sensed via electrode potentials or piezoresponse amplitude mapping. To enhance the locality and tunability of charge manipulation, the charge-driving electrode may be fabricated from a semiconducting material with partial screening, allowing for both global and locally addressable control. This configuration supports combined time- and space-multiplexed data handling, expanding the functional versatility of ferroelectric reservoirs for neuromorphic computing tasks.

In the present study, we focus on the intrinsic physical dynamics of the ferroelectric reservoir, which performs the major part of nonlinear preprocessing. Consequently, the associated readout layer can remain minimal, typically linear in subsequent computational implementations.

### Contactless interfaces

**Figure 7:**
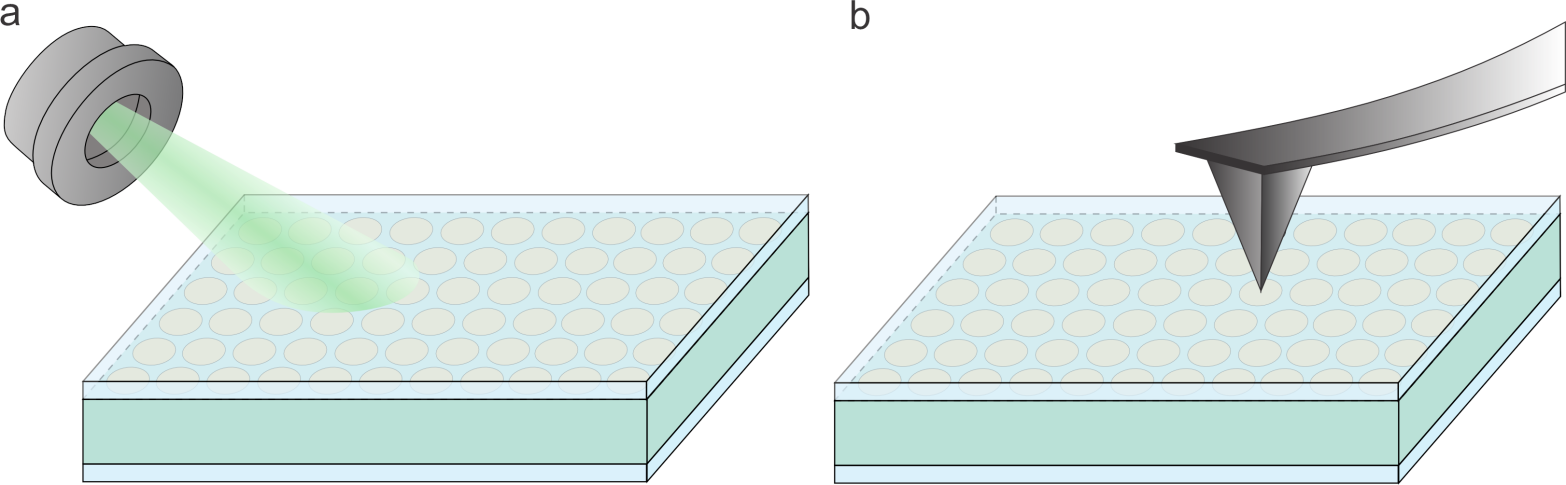
Contactless input and output methods for the ferroelectric reservoir. (a) Input and readout based on optoelectronic addressing and sensing. (b) Input and readout realized via atomic or piezoresponse force microscopy.

Contactless input and output methods of data integration between the reservoir and external neuromorphic interfaces are of particular importance for the development of scalable, high-resolution, ultrafast, and non-invasive neuromorphic systems. They enable direct interaction with the ferroelectric reservoir without physical wiring, expanding integration possibilities with optical platforms, scanning probe tools, and flexible electronics. In the implementations illustrated in Figure 7, the ferroelectric reservoir is operated using contactless methods for data input and output, where the array of ferroelectric nanodots serves as a pixel-wise data storage matrix.

In the configuration shown in Figure 7a, optoelectronic techniques are employed. At the input stage, a pixel-resolved optical beam locally excites charges at the top electrode, inducing spatially resolved switching of individual ferroelectric nanodots. The processed information, stored in the polarization states of the nanodots, is subsequently read out optically, again in a pixel-by-pixel manner. This approach enables fully contactless operation and is compatible with scalable, high-throughput optical interfaces.

An alternative configuration is shown in Figure 7b, where atomic force microscopy or piezoresponse force microscopy are used for both input and output operations. Here, the localized tip of the microscope is used to inject or sense charges at specific positions in the ferroelectric array, allowing for direct control or readout of the nanodots’ polarization states with nanoscale precision. Such techniques offer a high-resolution interface with the reservoir and can be particularly effective in prototyping, diagnostics, or specialized neuromorphic functions.

These contactless schemes are fully compatible with the broader architecture of ferroelectric reservoir computing and complement the time- and space-multiplexed electrical methods discussed in previous figures. Importantly, the ability to selectively write and read polarization states without electrodes enhances the potential for integrating the reservoir with optical systems, probe-based tools, or even hybrid analog-digital platforms.

## Conclusion

In this work, we have proposed and analyzed a ferroelectric-based data processing reservoir composed of nonlinear, bistable nanodots arranged in capacitor-like configurations. Each nanodot supports multiple polarization states with hysteretic switching, enabling robust information storage and processing. The assembled ferroelectric reservoir exhibits behavior governed by a frustrated Ising-type energy landscape, where the polarization configuration minimizes the electrostatic energy of the coupled system. Interaction matrix and local fields arise naturally from electrostatic coupling, while the discrete polarization states map to binary Ising variables. This analogy provides a physically grounded platform for implementing neuromorphic functionality.

The system operates through charge-controlled switching of polarization states, and its dynamic behavior depends not only on external inputs but also on prior polarization history. This results in noncommutative and path-dependent evolution trajectories, as demonstrated through examples involving two-, four-, and many-dot arrays. Particularly, sequential application of charge pulses leads to rich dynamics in high-dimensional configuration spaces, with the number of accessible metastable states growing combinatorially with the number of nanodots. Such complexity enables the encoding and transformation of temporal information in a manner suitable for reservoir computing architectures.

We demonstrated several architectures for signal injection and readout, including time- and voltage-driven schemes, spatially multiplexed control using localized electrodes, and contactless access via optoelectronic or scanning probe methods. These multiple input/output schemes offer a high degree of flexibility and compatibility with different system architectures. Notably, all implementations rely solely on dielectric polarization degrees of freedom, requiring no active current-driving elements. This makes the system particularly attractive for low-energy, high-density neuromorphic circuits.

The proposed ferroelectric reservoir benefits from several advantageous physical properties intrinsic to nanoscale ferroelectrics – high capacity, with exponentially growing configuration space, fading memory, provided by the slow relaxation dynamics of polarization in individual nanodots, intrinsic nonlinearity, stemming from the hysteretic switching behavior, and flexible integration, including time- and space-multiplexing schemes for efficient data exchange with synaptic layers.

As an extension of the proposed platform, the ferroelectric nanodots can be designed to host complex topological excitations, such as domains [45–46], vortices [47–49], helices [50], skyrmion-like structures [51–52], and hopfions [53], forming multistable polarization textures with nontrivial topology. These topological polarization configurations are not only stable against perturbations but can also support multilevel logic states beyond simple binary encoding. Their internal structure provides additional degrees of freedom that can be exploited for multivalued neuromorphic processing, enabling richer spatiotemporal dynamics and increased information density. Integration of topological excitations into the reservoir computing framework may further enhance robustness, programmability, and parallelism, opening up new opportunities for topologically protected logic and neuromorphic computation in future nanoscale devices.

## Data Availability

Data generated and analyzed during this study is available from the corresponding author upon reasonable request.
